# Learning an enriched representation from unlabeled data for protein-protein interaction extraction

**DOI:** 10.1186/1471-2105-11-S2-S7

**Published:** 2010-04-16

**Authors:** Yanpeng Li, Xiaohua Hu, Hongfei Lin, Zhihao Yang

**Affiliations:** 1 Department of Computer Science and Engineering, Dalian University of Technology, Dalian, Liaoning, 116024, China; 2 College of Information Science & Technology, Drexel University, Philadelphia, PA 19104, USA

## Abstract

**Background:**

Extracting protein-protein interactions from biomedical literature is an important task in biomedical text mining. Supervised machine learning methods have been used with great success in this task but they tend to suffer from data sparseness because of their restriction to obtain knowledge from limited amount of labelled data. In this work, we study the use of unlabeled biomedical texts to enhance the performance of supervised learning for this task. We use feature coupling generalization (FCG) – a recently proposed semi-supervised learning strategy – to learn an enriched representation of local contexts in sentences from 47 million unlabeled examples and investigate the performance of the new features on AIMED corpus.

**Results:**

The new features generated by FCG achieve a 60.1 F-score and produce significant improvement over supervised baselines. The experimental analysis shows that FCG can utilize well the sparse features which have little effect in supervised learning. The new features perform better in non-linear classifiers than linear ones. We combine the new features with local lexical features, obtaining an F-score of 63.5 on AIMED corpus, which is comparable with the current state-of-the-art results. We also find that simple Boolean lexical features derived only from local contexts are able to achieve competitive results against most syntactic feature/kernel based methods.

**Conclusions:**

FCG creates a lot of opportunities for designing new features, since a lot of sparse features ignored by supervised learning can be utilized well. Interestingly, our results also demonstrate that the state-of-the art performance can be achieved without using any syntactic information in this task.

## Background

With the exponential explosion of biomedical literature such as MEDLINE, developing automatic information extraction (IE) tools is essential for people to seek information more accurately and efficiently [1]. The task of protein-protein interaction extraction (PPIE) aims to extract interacting protein pairs from biomedical literature, which contributes to PPI network analysis and discovery of new functions of proteins. In recent years, it has attracted a lot of research interests [2-16] from different domains such as bioinformatics, natural language processing (NLP), and machine learning (ML). Bioinformatics researchers focus on constructing corpus [2,3], designing domain-specific rules for PPIE [4,5] and integrating the extraction results into PPI network analysis [6]. In NLP community, a lot of works focus on how to apply linguistic parsing to enhance the extraction performance [7-12,15,16]. People find that methods that incorporate syntactic parsing information can improve the extraction performance significantly and outperform methods that use only lexical information. In addition, most of these methods are in the framework of machine learning. Recent studies [10-12,16] show that integrating all the lexical and parsing features in a kernel-based supervised learning model achieves state-of-the-art performance on benchmark datasets. Since in supervised learning there exists a feature space for each kernel [17], these methods essentially represent each interacting protein pair and their contexts by a feature vector and the weights of features are learned from labeled training data.

However, these methods are restricted to obtain knowledge from limited amount of labeled data, and when the data size is small there is no sufficient information to assign proper weights to low-frequency or out-of-vocabulary (OOV) features (not in training data but in new examples). Another case is that many sparse features are not included in the original features set because they do not perform well in the experiments and filtered by system designers at the beginning. But are these discarded features really worthless? For example, usually multi-word expressions are not selected as features in NLP tasks due to data sparseness, but intuitively their discriminating ability is strong. Can we utilize them to get a higher performance?

Fortunately, the huge amount of biomedical texts available online has provided rich background knowledge for solving the data sparseness, where a lot of low-frequency features in training data will become informative. The goal of this study is to leverage unlabeled data to enhance the representation of local lexical features and make better use of sparse features for the PPIE task. The learning method is based on our recently proposed semi-supervised learning strategy- feature coupling generalization (FCG) [18,19]. Its core idea is to create new features from the co-occurrences of two special types of raw features: example-distinguishing features (EDFs) and class-distinguishing features (CDFs). EDFs are strong indicators for the current examples and CDFs are strong indictors for the target classes. Intuitively, their co-occurrences in huge unlabeled data can capture indicative information that could not be obtained from limited amount of labeled training data. We used this method to learn an enriched representation of entity names from 17GB unlabeled biomedical texts for a gene named entity classification (NEC) task [18] and found that the new features outperformed elaborately designed local lexical features.

The primary work of this study is to design proper EDFs and CDFs for the PPIE task and compare the performance of new features with local lexical features. In addition, using the experimental data we investigate the different performances of low-frequency features in supervised methods and FCG, and show that FCG can utilize the sparse features ignored by supervised learning to get a better result.

 The rest of the paper is organized as follows: we first give a brief introduction of FCG framework, and then describe in details our methods for PPIE including preprocessing, local lexical feature and FCD feature design. Next, we investigate the performance of various features in our experiment. Finally, we conclude with discussion and possible future improvement.

## Methods

In this section, we first give a brief introduction of the general framework of FCG semi-supervised learning and discuss why it can be applied to the PPIE task. Then we design a set of Boolean features from the lexical information of contexts and select different EDFs and CDFs based on the Boolean lexical features. We compare the performance of FCD features with lexical features and also combine the two feature types in the final system.

### The general framework of feature coupling generalization

Feature coupling generalization [18] is a framework for creating new features from two special types of features: example-distinguishing features (EDFs) and class-distinguishing features (CDFs). EDFs are intuitively defined as “strong indicators” for the current examples, and CDFs are “strong indicators” for the target classes. The relatedness degree of an EDF *f_e_* and a CDF *f_c_* estimated from the unlabeled data *U* is defined as feature coupling degree (FCD), denoted by *FCD* (*U*, *f_e_*, *f_c_*). The FCG algorithm describes how to convert FCDs into new features. The assumptions behind this idea are: 1) the relatedness of an EDF and a CDF provides indicative information for classifying the examples that generate the EDF. 2) Given more unlabeled data, more FCDs that cannot be obtained from labelled data can be generated from unlabeled data.

Assume that *F* = {*f*_1_, …, *f_n_*} is the feature vocabulary of “raw data” that contains every Boolean feature one could enumerate to describe an example, and **X** ⊆ **R^n^** is the vector space of the raw data, where each example is represented by a n-dimensional vector** x** = (*x_1_*, …, *x_n_*) ∈ **X**. The algorithm process of FCG [18] can be summarized as follows:

1) Select the “example-distinguishing” part of *F* as EDFs, denoted by *E* ⊆ *F*.

2) Map each element in *E* to a unique higher-level concept (EDF root) in the set *H*, denoted by *root (e)*: *E* → *H*.

3) Select the “class-distinguishing” part of *F* as CDFs, denoted by *C* ⊆ *F*.

4) Define the set of FCD types *T* to measure the relatedness of EDFs and CDFs.

5) Let the vocabulary of FCD features be *H* × *C* × *T* so that each FCD feature maps a tuple (*h*, *c*, *t*), where *h* ∈ *H*, *c* ∈ *C*, and *t* ∈ *T*.

6) Calculate FCDs from unlabeled data and convert each example from the old representation **x** to a new feature vector **x̃** by the equation:

             (1)

where* e*∈*E*, *x̃_i_* ∈ **x̃** ,* i* indexes each triple (*h, c, t*) in *H* × *C* × *T*. The operator *band*(*e*, **x**) (short for “Boolean And” operating) equals 1 if the feature *e* appears in the example **x**, and 0 otherwise.

For simplicity, here we assume that EDFs and CDFs are all extracted from *F*. In a broader sense, we can use the transformed feature set of original data to generate EDFs or CDFs. For example, the “CDF II” used in the NEC task [18] is the combination of local context words by a classifier. In the above algorithm, we assume *F* contains all the “feasible” combinations of original features derived from the data, and all the EDFs and CDFs are limited to be generated from this set.

In supervised learning, usually only a subset of elements in *F* can be utilized. This means features that don’t lead to performance improvement are treated as irrelevant ones, which are either removed before training or assigned very small weights during training to lower their impact. In FCG framework, we also need to select a subset of *F* as EDFs or CDFs, but the criterion for feature selection is rather different. Here ‘good’ EDFs or CDFs mean the performance of FCD features generated from them is good, although the performances of individual EDFs or CDFs might be poor in a supervised setting. In other words, irrelevant features in supervised learning may be good EDFs or CDFs that produce indicative FCD features, so that FCG could utilize the features ignored by supervised learning.

The selection of EDFs and CDFs plays a central part in this framework. We suggested that when selecting these features, a trade-off between “indicative” and “informative” should be considered [18]. In the NEC task [18] for determining whether an entity is a gene or protein name, the EDFs were selected as the whole entities and boundary word-level n-grams, and the CDFs were context patterns (such as “*X gene*” and “*the expression of X*”) and the discretized scores of a SVM trained by local contexts. The experiments show that good results can be achieved when various types of EDFs together with hundreds of CDFs are used. We also found that these FCD features performed better in non-linear classifiers than linear ones

The PPIE task can be treated as a binary classification problem that determines whether a sentence contains the evidence of the interaction of two focus proteins. It is natural to adapt the method to the PPIE task since we are just extended the method from “term classification” to “sentence classification”. However, there are two major differences: 1) there is position information of the two interacting proteins in each sentence, which makes it a little different from “sentence classification”. How can we incorporate the position information into EDFs or CDFs? 2) Usually there are more words in a sentence than in a named entity, which makes it difficult to use the whole sentence as EDFs since they are not informative even in the unlabeled data. How can we select the “example-distinguishing” part of a sentence that contains two proteins?

### Corpus and pre-processing

We used AIMED corpus to examine our methods because: 1) many previous works [2,7-13,15,16] were evaluated on this corpus. 2) It focuses on the single performance of relation extraction rather than BioCreative 2 PPI task [20], where the whole text mining process including protein name recognition, normalization, and PPIE are evaluated together.

Pyysalo *et al. *[21] made a comparative analysis of five PPI corpora and converted all the corpora [21] to a common format to facilitate research in this area. The recent work [10,16] was evaluated on this dataset. We used their transformed version of AIMED corpus, which contains 1000 positive and 4834 negative examples.

We converted each sentence to lowercase, replaced XML tags like “*&quot;*” by their standard ASCII characters, and then a sentence is tokenized by splitting tokens from non-letter or digit characters, e.g., “*wild-type (d)*” -> “*wild - type ( d )*”. We replaced the two focus proteins in the current example by “*prot1*” and “*prot2*”, and the other proteins in the same sentence by “*prot0*”. We also replaced all the examples with overlapping “*prot1*” and “*prot2*” by the same sentence “*prot1 prot2 .*” We ignored these self-interactions just for simplicity, since they will make it difficult to design a consistent feature set. The reason we kept these examples in the dataset is to keep the numbers of examples consistent with other works [10,16] on the same corpus for a fair comparison.

### Lexical information

As discussed in previous sections, in each example, the positions of the two focus proteins make the task different from a simple sentence classification task. We will show that they play an important role in our FCD and local lexical features. For clarification, we will give some notions of words, n-grams, areas and positions in a sentence with regard to two interacting proteins.

Vocabularies of words: LW = {*words in labeled data*}, and UW = {*words in unlabeled data*}.

Vocabularies of word-level n-grams: LN = {*1-3 grams in labeled data*}, and UN = {*1-3 grams in unlabeled data*}.

Assume that words in an example are indexed by the following sequence:

*Indices* = (0, …, *i1*, …, *i2*, …, *END*)

where *i1* is the index of the token “prot1”, *i2* is the index of “prot2”, and *END* is the index of the last token of the sentence.

General areas: GA = {*Left_Area*, *Inner_Area*, *Right_Area*} = {[0, *i1*-1], [*i1*+1, *i2*-1], [*i2*+1, *END*]} –text snippets split by “*prot1*” and “*prot2*” in each sentence denoted by “*Left_Area prot1Inner_Areaprot2 Right_Area*”.

Surrounding areas: SA = {*P1_Left*, *P1_Right*, *P2_Left*, *P2_Right*} = {[*i1*-4, *i1*-1], [*i1*+1, *i1*+4], [*i2*-4, *i2*-1], [*i2*+1, *i2*+4]} – texts surrounding “*prot1*” or “*prot2*” within a 4-word window.

Specific positions: SP = {*m_From_P1*, *n_From_P2* | *m* = *x* - *i1*, *n* = *x* - *i2*, *x* ∈ [*i1*-5, *i1*+5] ∪ [*i2*-5, *i2*+5]} – words or n-grams that appear in certain positions in *SA* with the window size 5, and *x* is the index of the current text. Note that the definition of SP is somewhat different from our most recent work [19]. Here we use a more comprehensive set to describe the specific term positions in each example and find it more effective in our experiments.

Conjunct positions: CP = {* P1_direction* ^ *P2_direction* ^ *distance* | *direction* ∈ {*Left, Right*}, *distance* = discretized (*i2-i1*) ∈ {*0,1, 2, 3, 4, 5, (6~7), (8~10), (11~15), (16~20), (21~30), (31~40), (40~)* } – conjunctions of partial elements (two areas adjacent each protein) in SP and the discretized word count between the two proteins.

### Local lexical features

We note that the lexical features used in the recent works [11,12,16] based on AIMED corpus only involved bag-of-words or simple variants. Here we attempt to enhance lexical-level representation by incorporating n-gram and position information and give a detailed evaluation of the contribution of each feature type. Four types of features are investigated in our work:

GA-BOW – bag of words: features derived from LW × GA, e.g., “*Word_In_Left_Area=expression*”. These features ignore word positions in the current area, which are almost as the same as features of the baselines used in the works [11,12,16].

GA-Lex – bag-of-n-grams: features from LN × GA. It simply enriches the bag-of-words representation by bigrams and trigrams.

SA-Lex –n-grams surrounding proteins: features from LN × SA, e.g., “*P1_Right=interacts with*”. They are used to highlight n-grams in the “indicating areas”, since intuitively features surrounding candidate protein pairs are more indicative.

SP-Lex – n-grams with specific offsets from two proteins: features from LN × SP, which gives the information of specific distances from the n-grams in SA to protein candidates, e.g., for the example “*…prot1 interacts with prot2…*”, some of SP-Lex can be“*1_From_P1=interacts*”, “*2_From_P1=with*”, “*-1_From_P2=with” and “2_From_prot1 =interacts with*”. It provides more specific information than SA-Lex.

Conjunct position n-grams (CP-Lex): features from LN × CP. The feature set is the conjunction of a subset of features in SP-Lex and the distance of two proteins. It can simultaneously capture the lexical information around both proteins and is thus more indicative than SP-Lex. The only problem is that they may suffer from data sparseness.

Our classifier for all the lexical features is SVM ^light^[14] with linear kernel and default parameters.

### EDF selection

For a fair comparison with local lexical features, some of the EDFs used in our experiments are derived from most discriminating ones of local lexical features and we also propose some new features that have never been used in relation extraction to our best knowledge. Three types of EDFs are reported in this work:

SP-EDF: features derived from UN × SP. It can be viewed as the extension of SP-Lex features to the vocabulary of UN. Obviously it has stronger discriminating ability than features derived from GA or SA. The set of EDF roots is GA but not SP because features derived from SP result in a much higher dimension in feature space, which will bring heavy computational burden. We do not use SA as EDF roots because the performance is slightly inferior to GA in our experiments.

CP-EDF: features derived from UN × CP. The set of EDF roots is {*P1_Left*, *P1_Right*} × { *P2_Left*, *P2_Right*}. Each feature is a pattern that connects two n-grams each of which is adjacent to a different protein. One CP-EDF can be viewed as a partial conjunction of two SP-EDFs and the distance of two proteins, so its discriminating ability will be stronger than SP-EDFs. This feature set is the same as that in our work [19].

DS(discretized similarity)-EDFs: the discretized similarity with the target examples. If the similarity between the source and target examples is over a certain threshold, the feature indexed by the target example will be set “true” for the source example. We used cosine similarity function in our experiment, which is defined as follow:

				(2)

where the vector **s** and **t** represent source and target examples respectively. Each vector is generated by a modified set of SP-EDFs. There are four modifications: 1) we do not limit the window size, because we want to use more information from the whole sentences and this will not add much computational cost rather than SP-EDFs and CP-EDFs. 2) We remove unigrams (single words) from the vectors, leaving only bigrams and trigrams. 3) We add the terms that appear in SA to each vector three times in order to improve their weights. 4) If the two examples have different distances of two proteins, the similarity score will be zero. We use these strict match schemes to improve the discriminating ability of the similarity function. The features are indexed by the set DSEDF={*SimWith_target_Over_threshold* | *target* ∈ {*all the examples for training and testing*}, *threshold* ∈ {*0.1, 0.2, 0.3, 0.5, 0.8*}}. For an example **x**, we defined one EDF root which generates EDFs in the set {*e* | *e* ∈ DSEDF, *target(e)*=**x**}. This means in FCG (see also Equation 1) we sum up all the EDF-CDF pairs where the target of EDFs are the same as the current example.

For example, there is a labelled example **x** and a unlabeled example **u**, and assume that:1) there is no good features in **x** , possibly due to extreme sparseness in training data, but there is a good features *c* in **u**. 2) The similarity between **x** and **u** is over 0.8 , indicating the two examples are very similar. Then for both **x** and **u** the value of the feature *SimWith_***x_***Over_0.8* is “true”. For **x** the feature is a strong discriminating feature that can distinguish it from others, and its co-occurrence with *c* in **u** can provide indicative information beyond the training data. An example of EDF generation is shown in Table 1.

**Table 1:** Examples of EDFs

Current example:	The results show that prot1 heterodimerizes with prot0 and prot2 in vivo , but it does not homodimerize to a measurable extent .
SP-EDF:	*-4_From_P1=the* *-9_From_P2=the* * -3_From_P1=results* *-8_From_P2=results* *-3_From_P1=the results* *-8_From_P2=the results* …, *1_From_P2=and prot2 in* ...

CP-EDF:	*P1_Left=that^P2_Left=and^5* *P1_Left=show that^P2_Left=prot0 and^5* *P1_Left=results show that^P2_Left=with prot0 and^5* *P1_Right= heterodimerizes^P2_Left=and^5* …

DS-EDF:	*SimWith_example0_ Over_0.8* *SimWith_example0_ Over_0.5* *SimWith_example0_ Over_0.3* *SimWith_example0_ Over_0.2* *SimWith_example0_ Over_0.1* *SimWith_example1_ Over_0.8* …

In SP-EDF, the distance of a protein and an n-gram is the word count between the protein and the last token of the n-gram. In the CD-EDF, the distance between the two proteins is 5, so all the features are joined with the distance.

### CDF selection

We used Chi-square – a popular feature selection technique – to rank local lexical features (GA+SA+SP) in labelled training data and selected top 400 ones as CDFs. This idea is similar to that in our former woks [18,19] where information gain was used. Here we find that the results obtained by Chi-square seem to capture more indicative patterns for PPIE. In Table 2, top ranked CDFs in our experiment are listed (see also the “Evaluation methods” section for the detailed implementation).

**Table 2:** Examples CDFs

CDFs	Chi-square	CDFs	Chi-square
*P2_Left=with* *P1_Right=with* *Inner_Area=with* *Left_Area=prot0* *-1_From_P2=with* *0_From_P2=with prot2* *2_From_P1=with* *Inner_Area=,* *2_From_P1=- prot2* *-1_From_P2=prot1 -*	219.34 194.41 183.05 168.05 118.53 118.53 106.68 106.62 93.10 93.10	*2_From_P1=prot1 - prot2* *0_From_P2=prot1 - prot2* *P1_Right=prot0* *Inner_Area=prot0* *P1_Right=interacts with* *-1_From_P2=between prot1 and* *P2_Left=between* *-3_From_P2=between* *-2_From_P2=between prot1* *P1_Left=binds*	93.10 93.10 83.61 83.09 80.94 79.3 75.91 74.4 74.4 68.17

Since the CDFs extracted from the training set of each round of ten-fold cross validation are highly overlapped, here we just list the top results of the first round. “Chi-square” is the score given by Chi-square feature selection method.

**Figure 1:**
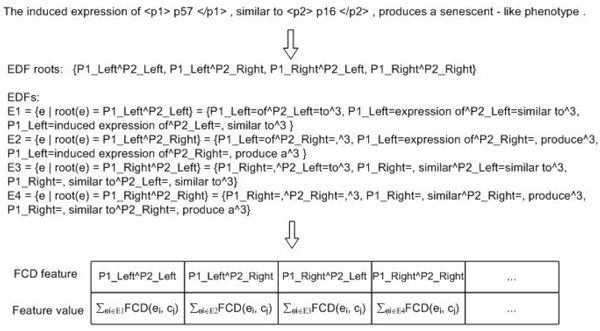
**An example that shows how FCG generates new feature for the PPIE task.** Here only CP-EDFs are considered. They are divided into four groups according to different EDF roots. A CDF is denoted by *c_j_*. Since only one FCD type is used here, the FCD features are indexed by the conjunction of EDF roots and CDFs.

Note that rather different from lexical features, these EDFs and CDFs are *not* elements of the input vectors of the target classifiers. They are used only for generating FCD features which belong to part of the final feature vectors. Figure 1 shows an example of the generation of FCD features for the PPIE task, where only SP-EDFs and one type of FCD measure are considered, so the FCD features are indexed by the conjunction of EDF roots and CDFs. It can be seen clearly that the sparse EDFs are generalized to a higher-level representation.

### Classifier

In the work [18], we found the density of FCD features was much higher than lexical features widely used in NLP and was somewhat like the feature spaces for image recognition, which inspired us to make use of non-linear classifiers. We used SVD plus RBF kernel and achieved better results than linear kernel. Similarly, for the PPIE task we also investigated the two models: linear SVM and RBF kernel based SVM. For the RBF model, we first used SVD to get a sub-space of FCD features and then used the new features as the inputs of SVM with RBF kernel. In our experiments, SVD was done on the entire AIMED corpus and top 200 most significant features in left-singular matrix were selected. The parameter “-c” and “-g” of SVM^ light^ were set at 3 and 20. Then we combined the prediction scores of lexical features and FCD features given by SVMs using a simple weighted linear function, where their weights were set at 3/5 and 2/5 respectively.

### FCD measure & unlabeled data

In this work, we consider one type of FCD measure:

                    (3) 

where *x* is an EDF, *y* is a CDF, and *co(x, y)* is the co-occurrence count of *x* and *y*. The smoothing factor *b* is assigned 1. We log the term count to avoid highly biased values in very large corpus. This measure can be viewed as a variant of pointwise mutual information (PMI). We discussed its advantage in the work [18].

The experimental results in [18] show that the performance of FCD features increases when more unlabeled data are added. So in this work, we downloaded more data, which include all the PubMed abstracts (up to 2009) and data collection of TREC genomics track 2006 [22,18], with the total size of 20GB. We tokenized the texts using the same method as introduced previously and tagged the protein names using the gene/protein mention tagger developed in the work [18]. We used the “dictionary-based” method because it is very fast (over 10,000 sentences per seconds). The method for the dictionary construction was also based on FCG and it achieved an F-score of 86.2 on BioCreative 2 Gene Mention test corpus [23]. Note that in the PPIE task, “unlabeled” means no need to label the protein-protein interactions, but the protein names should be recognized first. For efficiency we removed the sentences that contain over 10 protein names in the experiments. Finally we obtained around 47 million unlabeled examples.

## Results and discussion

### Evaluation methods

Although AIMED corpus was used for evaluation by many researchers, it is still difficult to compare their results exactly, because they used different evaluation, data pre-processing and training/test set splitting methods in their experiments. We try to keep our evaluation metrics as the same as the recent works [10-12,16]. We used F-score as the primary evaluation measure and also reported AUC. Airola *et al. *[10] suggested that for this task, abstract-level cross validation should be used to avoid sentences in the same abstract are both used for training and testing. We also performed abstract-wise 10-cross validation, where abstracts were divided into 10 groups, and one was used for testing and the others for training in each round. We extracted CDFs from each training data separately to avoid the use of answers in test set at training time.

### 4.2. Local lexical features

Table 3 shows the performances of various combinations of local lexical features. We can see the F-score of GA-BOW features is 50.17, which is similar to the results reported in the recent work [11], where the F-score of a similar feature set is 51.1. The discrepancy may be caused by lemmatization they used, or the detailed methods in data preprocessing and splitting stages. It can be seen that features derived from surrounding areas and specific position information improve the performance significantly and produced a surprisingly good result – 61.4 F-score and 86.11 AUC, which is better than most of the recent works based on syntactic parsing [10,11] (see also “Comparison with other systems” section). Note that this run only used simple Boolean lexical features, so it is much faster and easier to implement than syntactic based methods, which will make it feasible in practice. To our best knowledge, the features (F2, F3, F4 and F5) are not explicitly used as Boolean lexical features in the PPIE task and their contribution is not examined well on the AIMED corpus. The simple idea of creating these lexical features is similar to our work on entity classification [18], just following the cue from general to concrete: “word -> n-grams -> n-grams in specific positions”.

**Table 3:** Performance of local lexical features

Feature	Precision	Recall	F-score	AUC
F1	42.2	**65.12**	50.17	78.22
F1+F2	45.83	61.65	50.89	78.92
F1+F2+F3	54.06	60.25	56.39	83.1
F1+F2+F3+F4	60.61	63.43	61.11	85.97
F1+F2+F3+F4+F5	**62.13**	62.4	**61.4**	**86.11**

F1: GA-BOW; F2: GA-Lex; F3: SA-Lex; F4: SP-Lex; F5: CP-Lex

From Table 3, the additional contribution of bigrams and trigrams over unigrams seems not big. We will analyze the reason in the “Sparse features in FCG and supervised learning” section together with FCD features and show it is mainly due to the data sparseness in training corpus.

### FCD features

Table 4 lists the performances of various types of FCD features. It can be seen that among FCD features the method that integrates all the features in RBF kernel achieves the best result – a 60.06 F-score and an 83.78 AUC. The combination of FCD and lexical features achieve a 63.54 F-score and an 87.24 AUC. It improves the performance of lexical features by over 2% in F-score, but the improvement is a little lower than our results in the work [19], it is because the modified SP features here lead to significant improvement to SP-EDFs and SP-Lex, but not so much for the combination of FCD features.

**Table 4:** Performance of FCD features

ID	Local	SP-EDF	CP-EDF	DS-EDF	Linear	RBF	Precision	Recall	F-score	AUC
1	*				*		**62.13**	62.40	61.4	86.11
2		*			*		53	64.48	57.6	81.75
3		*				*	58.08	62.67	59.16	82.97
4			*		*		56.83	53.55	54.33	79.26
5			*			*	54.64	57.71	54.47	78.34
6				*	*		53.5	56.4	53.87	78.02
7				*		*	54.45	58.71	55.56	79.79
8		*	*	*	*		54.08	63.66	58.06	82.61
9		*	*	*	*	*	59.7	61.52	60.06	83.78
10	*	*	*	*	*	*	60.47	**68.31**	**63.54**	**87.24**

In RBF SVM, SVD is used to reduce the feature dimension and a SVM with RBF kernel is used to classify examples. Run 10 combines the outputs of Run 1 and Run 9.

From Table 4, it also can be seen that of all the single types of features SP-EDF perform best, possibly because the n-grams with specific offsets are both indicative and informative in the unlabeled data compared with other EDFs. CP-EDFs are the conjunction of two SP-EDFs and thus more discriminating, but the performance is 5 percent lower than SP-EDFs. We believe the reasons: 1) we just select a subset of SP for the conjunction due to the limitation of computational complexity. 2) Sufficient co-occurrences of CP-EDF and CDFs cannot be obtained from our unlabeled data, even though the data size is big. It seems that DS-EDFs are able to find a good trade-off between indicative and informative, but its performance is still inferior to SP-EDFs. It indicates there may be other factors that determine the quality of EDFs, such as a better similarity function between examples that can reflect their “real similarity” under this task. In addition, there are two common reasons that can explain the inferior performance of CP-EDFs and DS-EDF: 1) different EDFs may prefer different CDFs, but we fix CDFs in these experiments, which limits the full use of some EDFs. 2) In SP-EDF, the number of EDFs per example is more than CP-EDFs and DS-EDFs, which show that the combination of weak EDFs can beat strong ones. This analysis can inspire us to investigate and quantify the factors that determine the quality of EDFs and CDFs, so that automatic selection of better FCD features will become realistic in the future.

From Table 4, we can see that the performance of SVD-RBF model is at least as good as linear model, which is a similar to our previous work on entity classification [18]. It is an important finding for NLP community, since we find a general way to generate a non-linear representation for natural language which has at least as good performance as linear methods that have been widely used in machine learning for NLP. In our experiments, we just examined a simple non-linear method, and we believe that there will be a better non-linear model for this task or FCD features in general.

**Figure 2:**
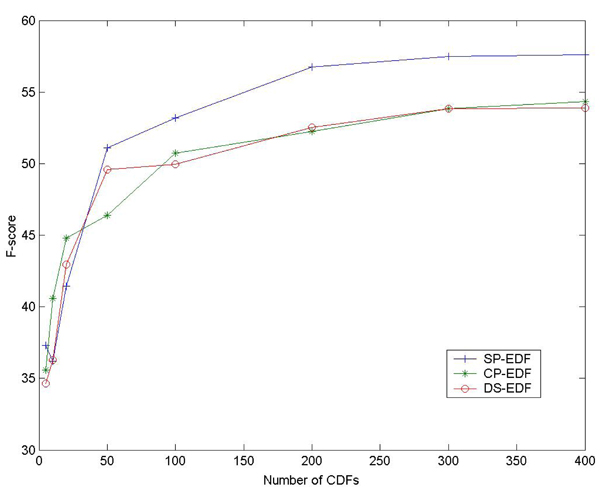
**Relation between the performance of FCD features and the number of CDFs.** The patterns are selected in a descendent order of Chi-square scores. For all the FCD features, linear classifiers are used.

**Figure 3:**
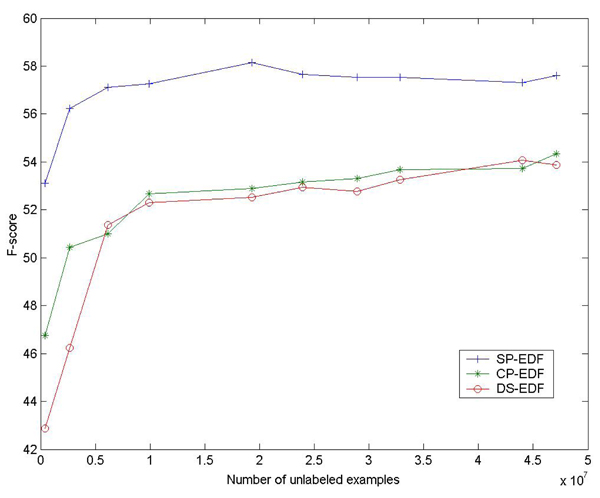
**Relation between the performance of FCD features and the number of unlabeled examples.** Here linear classifiers are used.

Figure 2 and Figure 3 show the relation between the performances of FCD features, the numbers of CDFs and scale of unlabeled examples. Similar to our earlier results on entity classification [18], generally there is consistent improvement when more CDFs and unlabeled data are incorporated. So in the future we may consider using more CDFs and unlabeled data to enhance the performance. But we think it a more important work to investigate what kind of CDFs or unlabeled data can contribute more to the performance of FCD features.

### Comparison with other systems

In Table 5, we compare the performances of our methods with other results reported in previous researches evaluated on AIMED corpus. Although it is difficult to make a strict comparison due to different methods for data splitting and pre-processing, it can be seen that our combined method is among the state-of-the-art systems. It is an important finding for both biomedical text mining and NLP community, because unlike other methods, no syntactic information is used in this run. But it doesn’t mean there is conflict between FCG and syntactic features and we can also consider incorporating syntactic information into FCG or just combining our results with others to get higher performance in the future. Another interesting finding is that our baseline with simple local lexical features not only achieves good results but is much more efficient and robust than syntactic based methods.

**Table 5:** Comparison with other systems on AIMED corpus

Method or author	F-score	AUC
(Miwa et al., 2009) [16]	65.2	89.3
Our method (Combined)	63.5	87.2
(Miwa et al., 2009) [12]	62.7 (64.3)	83.2 (87.9)
Our method (Lex)	61.4	86.11
Our method (FCD)	60.1	83.8
(Miyao et al., 2008) [11]	59.5	-
(Airola et al., 2008) [10]	56.4	84.8
(Sætre et al., 2007) [9]	52.0	-
(Mitsumori et al., 2006) [14]	47.7	-

For the work [12], the scores in brackets are their reported results obtained by removing all the examples with self-interaction protein pairs in AIMED corpus.

In the work [18], we discussed the efficiency of FCG in real world applications. In summary, for real-time application, it needs the support of “feature-level” search engine. Alternatively if the task can be divided into non-real-time sub-tasks, we can run FCG on each sub-task in an offline manner. For example, in this task, we can generate a huge number of lexical patterns indicating for PPI and used FCG to remove noisy patterns. Then the refined patterns are used as features integrated into the lexical feature-based method. The idea is similar to the dictionary construction for the NER task [18]. In this way, we can utilize the information from unlabeled data and make the system efficient.

### Sparse features in supervised learning and FCG

**Figure 4:**
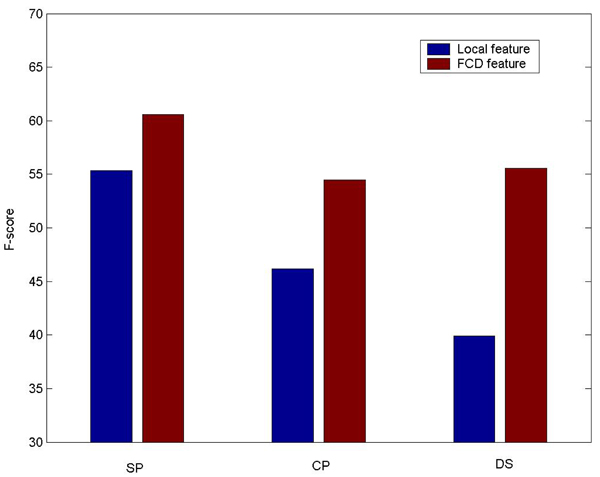
**Comparison of local lexical features and FCD features.** For FCD features, non-linear classifiers are used.

In this section we try to answer the question discussed in the “Background” section: how can FCG utilize the sparse features ignored by supervised learning? Using the data in our experiments, we observed very interesting results. Figure 4 shows the performance comparisons of local lexical features and FCD features derived from similar lexical information. It can be seen that FCD features perform much better than lexical features consistently. We note that the densities of features derived from SP, CP and DS in the AIMED corpus are respectively 0.05%, 0.023% and 0.025%. Therefore the results seem to show that when the features are extremely sparse in training data, the performance will be poor in supervised learning, but can be enhanced by FCG from huge unlabeled data.

**Figure 5:**
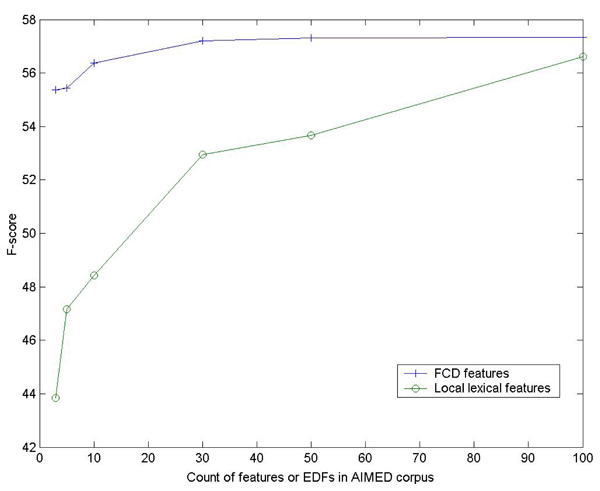
**Sparse features in FCG and supervised learning** The *x* axis is the count of features (EDFs for FCD features) in the AIMED corpus. The* y* axis is the F-score obtained by training with all the features equal or less than the certain feature count indicated by *x* axis. For FCD features, linear classifiers are used.

More results can be found in Figure 5, which shows the performance of different feature sets when only features with less than certain counts in AIMED corpus are used. As can be seen, when using the extremely sparse features (e.g., less than 4 times in AIMED corpus), the performance of local features are rather poor, but there is no big impact on FCD features. We also found that when these features were removed from local lexical features, there was almost no negative impact on the performance, which indicates that these features are ignored in supervised learning. It is encouraging to see that these discarded features can be good EDFs and perform well in FCG together with the huge unlabeled corpus. We obtain two important conclusions from the results: 1) since FCG can activate the inactive features in supervised learning, the room for designing new features will become much larger and a lot of features that have never been used in NLP can be examined, such as DS-EDFs in our experiments. 2) It inspires us to develop automatic methods for selecting EDFs concerned with the frequency of features, since the results show that FCG seems to prefer features with high sparsity, which is opposite to supervised learning.

## Conclusions

We present the application of FCG semi-supervised learning strategy to the PPI extraction task and show that FCD features derived from simple lexical information can achieve good results and produce further improvement over a high baseline. We believe that there is still a lot of room for improvement. In the future work, we will focus on designing EDFs and CDFs that cover more lexical or linguistic information (e.g., from shallow or syntactic parsing) of the whole sentences. Since many experiments show that FCD features perform well in non-linear classifiers, we will examine other popular learning techniques in pattern recognition. It is encouraging to see that FCG performs well in the two different NLP tasks: entity classification and relation extraction, so we will continuously examine this method in more tasks on natural language processing and machine learning.

The results also indicate that the frequency of features seems to be an indicator for the quality of EDFs and we believe that there must be other factors that determine the quality of FCD features. In the future work, we will investigate these impacts through experiments as well as theoretical analysis on different datasets.

Major contributions of this work can be summarized as follows:

1) We are the first to apply FCG to the task of PPIE. We proposed several novel types of EDFs/CDFs and got promising results on a popular dataset.

2) Our work create much more opportunities for designing new features for this task, since the results show that a lot of sparse features ignored by supervised learning can work well in FCG.

3) Our methods indicate that state-of-art results can be achieved without using any syntactic information in the PPIE task or even in general relation extraction tasks.

4) Our results demonstrate that semi-supervised learning can work well on huge amount of unlabeled data in this task.

5) We analyze different performance of features in supervised and semi-supervised learning with regard to the sparsity of features and obtain some interesting findings.

## Competing interests

The authors declare that they have no competing interests.

## Authors' contributions

YL conceived the idea, designed the experiments, and drafted the manuscript. XH, HL and ZY guided the whole work and helped to revise the manuscript. All authors have read and approved the final manuscript.

## References

[B1] Cohen AM Hersh WR A survey of current work in biomedical text mining. Briefings in Bioinformatics 2005 6 57 71 10.1093/bib/6.1.57 15826357

[B2] Bunescu R Ge R Kate R Marcotte E Mooney R Ramani A Wong Y Comparative Experiments on Learning Information Extractors for Proteins and their Interactions. Artificial Intelligence in Medicine 2005 33 2 139 155 10.1016/j.artmed.2004.07.016 15811782

[B3] Pyysalo S Ginter F Heimonen J Björne J Boberg J Järvinen J Salakoski T BioInfer: A Corpus for Information Extraction in the Biomedical Domain. BMC Bioinformatics 2007 8 50 17291334 10.1186/1471-2105-8-50 PMC1808065

[B4] Corney DP Buxton BF Langdon WB Jones DT BioRAT: extracting biological information from fulllength papers. Bioinformatics 2004 20 3206 3213 10.1093/bioinformatics/bth386 15231534

[B5] Fundel K Kuffner R Zimmer R RelEx-Relation extraction using dependency parse trees. Bioinformatics 2007 23 3 365 371 10.1093/bioinformatics/btl616 17142812

[B6] Hu X Wu D Data mining and predictive modeling of biomolecular network from biomedical literature databases. IEEE/ACM Transactions on Computational Biology and Bioinformatics (TCBB) 2007 4 2 251 263 10.1109/TCBB.2007.070211 17473318

[B7] Giuliano C Lavelli A Romano L Exploiting Shallow Linguistic Information for Relation Extraction From Biomedical Literature. Proceedings of the 11th Conference of the European Chapter of the Association for Computational Linguistics 2006

[B8] Bunescu R Mooney R A shortest path dependency kernel for relation extraction. Proceedings of Human Language Technology Conference and Conference on Empirical Methods in Natural Language Processing Association for Computational Linguistics 2005 724 731

[B9] Sætre R Sagae K Tsujii J Syntactic features for protein-protein interaction extraction. Second International Symposium on Languages in Biology and Medicine 2007 short papers

[B10] Airola A Pyysalo S Björne J Pahikkala T Ginter F Salakoski T All-paths Graph Kernel for Protein-protein Interaction Extraction with Evaluation of Cross-corpus Learning BMC Bioinformatics 2008 9 Suppl 11 S2 10.1186/1471-2105-9-S11-S2 19025688 PMC2586751

[B11] Miyao Y Sætre R Sagae K Matsuzaki T Tsujii J Evaluating Contributions of Natural Language Parsers to Protein-Protein Interaction Extraction Bioinformatics 2009 25 3 394 400 10.1093/bioinformatics/btn631 19073593 PMC2639072

[B12] Miwa M Sætre R Miyao Y Ohta T Tsujii J Combining Multiple Layers of Syntactic Information for Protein-Protein Interaction Extraction. Proceedings of the Third International Symposium on Semantic Mining in Biomedicine 2008 101 108

[B13] Bunescu R Mooney R Subsequence Kernels for Relation Extraction Advances in Neural Information Processing Systems 18 MIT Press 2006 171 178

[B14] Mitsumori T Murata M Fukuda Y Doi K Doi H Extracting Protein-Protein Interaction Information from Biomedical Text with SVM. IEICE - Transactions on Information and Systems 2006 E89D 8 2464 2466 10.1093/ietisy/e89-d.8.2464

[B15] Van Landeghem S Saeys Y Peer Y Van de De Baets B Extracting Protein-Protein Interactions from Text using Rich Feature Vectors and Feature Selection. Proceedings of the Third International Symposium on Semantic Mining in Biomedicine 2008 77 84

[B16] Miwa M Sætre R Miyao Y Tsujii J A Rich Feature Vector for Protein-Protein Interaction Extraction from Multiple Corpora Proceedings of the 2009 Conference on Empirical Methods in Natural Language Processing 2009 1 1 121 130

[B17] Taylor J Cristianini N Vijay Kernel methods for pattern analysis 2004 Cambridge Univ. Press

[B18] Li Y Lin H Yang Z Incorporating Rich Background Knowledge for Gene Named Entity Classification and Recognition BMC Bioinformatics 2009 10 223 10.1186/1471-2105-10-223 19615051 PMC2725142

[B19] Li Y Lin H Yang Z Applying Feature Coupling Generalization for Protein-Protein Interaction Extraction 2009 IEEE International Conference on Bioinformatics and Biomedicine (BIBM 2009) 396 400

[B20] Krallinger M Leitner F Rodriguez-Penagos C Valencia A Overview of the Protein-Protein Interaction Annotation Extraction Task of BioCreative II Genome Biology 2008 9 Suppl 2 S4 1 10.1186/gb-2008-9-s2-s4 18834495 PMC2559988

[B21] Pyysalo S Airola A Heimonen J Björne J Ginter F Salakoski T Comparative Analysis of Five Protein-protein Interaction Corpora BMC Bioinformatics 2008 9 Suppl 3 S6 10.1186/1471-2105-9-S3-S6 18426551 PMC2349296

[B22] Hersh W Cohen A Roberts P Rekapalli HK TREC 2006 genomics track overview. Proceedings of 15th Text REtrieval Conference (TREC) 2006

[B23] Wilbur J Smith L Tanabe L BioCreative 2. gene mention task. Proceedings of the Second BioCreative Challenge Evaluation Workshop 2007 7 16

